# PReS-FINAL-1005: Hypertransaminasemia in systemic juvenile idiopathic arthritis during anti-interleukin 1 treatment

**DOI:** 10.1186/1546-0096-11-S2-P3

**Published:** 2013-12-05

**Authors:** MI Gonzalez Fernandez, R Bou, R Silvia, E Iglesias, J Sánchez, V Torrente, J Antón

**Affiliations:** 1 Paediatric Rheumatology Unit. Pediatrics Department, Hospital Saint Joan de Déu, Esplugues, Barcelona, Spain

## Introduction

Systemic juvenile idiopathic arthritis (sJIA) accounts for 10-15% of JIA patients and is characterized by arthritis with fever, plus rash, generalized lymphadenopathy, hepatosplenomegaly and serositis. It is associated with significant morbidity and may be complicated with macrophagic activation syndrome (MAS). The use of anti-interleukin 1 (IL1) therapy results in dramatic improvement in both the systemic and articular disease.

Elevation of liver enzymes can be seen in sJIA patients because of disease activity, MAS, an infection, hepatotoxicity from pharmacologic treatment or as manifestation of another disease (such as autoimmune hepatitis). Achieving the diagnosis of the hypertransaminasemia in these patients may be difficult for the clinician.

## Objectives

To report 4 patients with sJIA who presented significative and difficult to interpretate hypertransaminasemia while being on IL1 blockers.

To identify and review other sJIA patients in our unit with hypertransaminasemia during anti-IL1 treatment.

## Methods

Medical reports from the sJIA patients treated with IL1 blockers in our unit were reviewed.

## Results

8 patients out of 30 sJIA patients treated with IL1 blockers in our Pediatric Rheumatology Unit since 2004, presented elevation of liver enzymes during anti-IL1 treatment. In 4 of these 8 patients, hypertransaminasemia was thought to be related with disease activity or secondary MAS. The other 4 cases were more difficult to interpretate.

Patient 1 presented elevated transaminases (ALT 2254 UI/L) when being 11 months on anakinra (initiated at disease onset), while prednisone tapering 40 days after a MAS. ASMA 1/80. IgG elevation. No evidence of disease flare, MAS, infection, nor other systemic diseases with liver involvement. Anakinra was stopped and liver enzymes decreased. Liver biopsy: compatible with autoimmune hepatitis. Good response to zathioprine. Anakinra was re-started 4 months later because of a disease flare without subsequent transaminases elevation. Patients 2 and 3 presented ALT 1924 UI/L and 852 UI/L respectively after 3 weeks on anakinra. The workup of hepatitis did not identify a cause. Anakinra was stopped with liver enzymes normalization within 2 months. Patient 4 is a persistent activity patient steroid dependent requiring different drugs during her follow-up. She presented liver enzymes elevations after one dose of canakinumab, while being on anakinra, but also after stopping them; maximum ALT 560 UI/L. Liver biopsy: inflammatory infiltrate without fibrosis. ASMA 1/80. IgG elevation. No evidence of infection, nor other disease with liver involvement. Now she is on anakinra, azathioprine and prednisone with normal liver tests.

## Conclusion

IL1 inhibitors are effective in sJIA.

Hypertransaminasemia in sJIA patients may result a challenge for the clinician.

The possibility of being related to anti-IL1 treatment should be taken into account. Three other cases of hypertransaminasemia in sJIA patients during treatment with IL1 antagonist have been reported, suggesting that a close monitoring for hepatic toxicity may be indicated when treating with IL1 blockers.

Autoimmune hepatitis is another diagnostic possibility, as shown in two of our patients.

## Disclosure of interest

None declared.

